# Effects of *In Vitro* Exposure to Diarrheic Toxin Producer *Prorocentrum lima* on Gene Expressions Related to Cell Cycle Regulation and Immune Response in *Crassostrea gigas*

**DOI:** 10.1371/journal.pone.0097181

**Published:** 2014-05-13

**Authors:** Reyna de Jesús Romero-Geraldo, Norma García-Lagunas, Norma Yolanda Hernández-Saavedra

**Affiliations:** 1 Molecular Genetics Laboratory, Centro de Investigaciones Biológicas del Noroeste, S.C. La Paz, Baja California Sur, México; 2 Department of Engineering, Instituto Tecnológico de La Paz, Baja California Sur, México; Veterinary Pathology, Switzerland

## Abstract

**Background:**

*Crassostrea gigas* accumulates diarrheic shellfish toxins (DSP) associated to *Prorocentrum lima* of which Okadaic acid (OA) causes specific inhibitions of serine and threonine phosphatases 1 and 2A. Its toxic effects have been extensively reported in bivalve mollusks at cellular and physiological levels, but genomic approaches have been scarcely studied.

**Methodology/Principal Findings:**

Acute and sub-chronic exposure effects of *P. lima* were investigated on farmed juvenile *C. gigas* (3–5 mm). The Pacific oysters were fed with three dinoflagellate concentrations: 0.3, 3, and 30×10^3^ cells mL^−1^ along with a nontoxic control diet of *Isochrysis galbana*. The effects of *P. lima* on *C. gigas* were followed by analyzing expression levels of a total of four genes, three involved in cell cycle regulation and one in immune response by polymerase chain reaction and real time quantitative PCR, where changes in time and cell concentration were found. The highest expression levels were found in oysters fed 3×10^3^ cells mL^−1^ at 168 h for the cycle regulator *p21* protein (9 fold), chromatin assembly factor 1 *p55* subunit (8 fold), elongation factor 2 (2 fold), and lipopolysaccharide/β-1, 3 glucan binding protein (13 fold above base line). Additionally, the transcript level of all the genes decreased in oysters fed wich the mixed diet 30×10^3^ cells mL^−1^ of dinoflagellate after 72 h and was lowest in the chromatin assembly factor 1 *p55* subunit (0.9 fold below baseline).

**Conclusions:**

On *C. gigas* the whole cell ingestion of *P lima* caused a clear mRNA modulation expression of the genes involved in cell cycle regulation and immune system. Over-expression could be related to DNA damage, disturbances in cell cycle continuity, probably a genotoxic effect, as well as an activation of its innate immune system as first line of defense.

## Introduction

Bivalve mollusks accumulate toxins during harmful algal blooms (HABs) making them vectors that pose a health hazard to humans who consume them [1], [2]. Shellfish contamination by algal toxins is one of the most serious problems for aquaculture and fisheries industries worldwide [3] causing major economic losses and bad publicity for seafood as a food resource [1], [4], [5]. HAB biotoxins have been widespread in European coasts where most notably diarrheic shellfish poisoning (DSP) toxins have been documented and studied. Due to their frequent presence, the DSP syndrome is now a global disease [6], [7].

DSP toxins are a type of acidic polyether toxins that include okadaic acid (OA) and its derivatives known as dinophysistoxins (DTX1, DTX2) and DTX3 [7], [8], which are characterized by a rapid onset of gastrointestinal symptoms in humans, such as vomiting and diarrhea, generally resolving within 2–3 days [7]. The main OA effect is the specific inhibition of serine and threonine phosphatases 1 (PP1) and 2A (PP2A) resulting in hyperphosphorylation of many cell proteins [9]. Since the number of physiological processes in which these phosphatases are involved is immense [10], the potential effects of OA are critical for cell development because it binds to the catalytic subunit and inhibits its enzymatic activity. The potentially affected proteins are intracellular components that signal transduction pathways in eukaryotic cells, which in turn regulate a diverse array of processes involved in metabolism, ion balance, neurotransmission, and cell cycle regulation (including metabolism regulation and gene expression) where reversible phosphorylation of their components is a major regulatory mechanism to control their activities [11].

The DSP causative organisms are dinoflagellates of the genera *Dinophysis* and *Prorocentrum* [12], [13]. *P. lima*, which has been commonly found in the Gulf of California, Mexico [14], is a toxic, benthic, and epiphytic dinoflagellate responsible for red tides in many localities along the Mexican Pacific coast where the presence of DSP in humans has been frequently reported [15], [16], [17].

Although death incidences due to OA poisoning have not been reported, and its toxic potency is much lower intraperitoneally in mice than that of polyether neurotoxin (LD_50_ = 192 µg/kg) [18], this molecule has been identified as tumor promoter [19] and apoptosis inductor [20]. Indeed, OA acts as a cytostatic drug by interfering with the control and expression of cell cycle regulatory proteins. In fact, OA potential to modify these proteins led to speculate that it might function as an exogenous mitogenic growth factor [21]. Therefore, gene expression related to cell cycle and its functional status, either inhibition or induction, can serve as a biomarker to understand and determine hazardous biotoxin effects in marine habitats.

Despite shellfish appear to be only toxin vectors unaffected by HABs, some bivalve behavioral, physiological, and cellular responses to *Prorocentrum* have already been described [6], [22]. The effects of microalgal toxic on bivalves have been studied through ingestion, absorption and accumulation rate; DSP toxins are accumulated mainly on digestive gland [22], [23], [24]; filtration activity reduction, pseudo-feces production, oxygen consumption changes, and generalized tissue inflammation principally of digestive organs [1], [4], [23], [24], [25]. An impairment of larval survival and reproductive development anomalies [26] and increases on the lysosomal destabilization in oysters’ hepatopancreas have been observed [27].

Recently, *in vitro* assays have shown that HAB species such as *Karenia brevis* (brevetoxin producer) [28], *Dinophysis acuminata* [29], *Alexandrium* sp. (PSP toxin producer) affect viability and phagocytosis in bivalves’ immune cells significantly [30]. Consequently studying the effects of harmful algae on bivalves’ immune system has recently become an area of great interest for researchers; various publications have demonstrated that hemocytes, as well as immune parameters, may be activated or modulated under the presence of several species of toxic microalgae [31], [32].

However, few studies have addressed gene expression changes in *C. gigas* in response to toxic algal exposure or to their toxins. Currently, a mussel cDNA digestive gland microarray fed for five weeks with OA contaminated nutrient reported a general up-regulation of transcripts coding for stress proteins and those involved in cellular synthesis [33]. The Pacific oyster *C. gigas* is a suspension-feeding bivalve mollusk, of great interest as a study model given its ecological, economic, public health, and genomic relevance because it has a completely sequenced genome [34], [35]. The objective of our study was to determine the effects of *P. lima* on farmed *C. gigas* juveniles (3–5 mm), which were investigated by both acute (0, 3, 6, and 24 h) and sub-chronic (72, 168, and 336 h) exposure. Four genes in total were monitored such as the cycle regulator *p21* protein (*Cg-p21*), chromatin assembly factor 1 *p55* subunit (*Cg-CAFp55*), elongation factor 2 (*Cg-EF2*), and lipopolysaccharide/β-1, 3 glucan binding protein (*Cg-LGBP*).

## Results and Discussion

### Feeding Response

**Figure 1:**
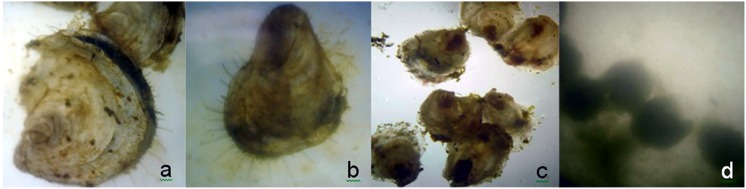
Images of pseudofeces produced by *Crasssostrea gigas* in the experimental bioassays. (a) Control diet *Isochrysis galbana* (0.75×10^6^ cells mL^−1^); (b) T1(0.3×10^3^ cells mL^−1^); (c) T2(3×10^3^ cells mL^−1^); (d) T3(30×10^3^ cells mL^−1^).

*Crassostrea gigas* exposed to toxic dinoflagellate cells showed immediate behavioral changes after consuming *P. lima* (strain PRL-1) [36], which consisted of partial shell-valve closure and pseudofeces and mucus production (Figure 1). However, pseudofeces were not feasible to measure under our experimental conditions because the experimental units had an aeration system that causes disintegration on feces and pseudofeces; besides small oysters’ susceptibility to stress by manipulation may overestimate the challenge response.

During the 0–3 h time frame, treated oysters filtered moderate quantities of the toxic dinoflagellate with partial shell-valve closure; however, after a 6 h exposure, oysters appeared to filtrate normally. Oysters showed adaptability strategies to cell density augmentation in water, such as increasing their filtration rate and/or producing pseudofeces. Oysters’ ingestive adaptability, as well as pseudofeces production has been shown as a major pre-ingestive mechanism because it not only prevents exceeding its ingestive capacity but also facilitates the process of particle selection, whereby less nutritious particles may be rejected and the quality of ingested material could improve proportionately [37], [38].

In research conducted by our group, we have observed immediate changes in feeding behavior after the contact with toxic dinoflagellates *P. lima* and *Gymnodinium catenatum*, as well as changes on immediate stress response gene expressions under acute and sub-chronic exposure, such as GS (glutamine syntetase), GST (glutathione S transferase and HSP_70_ (heat shock protein 70) [39], [40] and Cu/Zn-SOD [40] that showed to be dose-time-dependent.

### Gene Expression Analysis by RT-PCR

The impact of *P. lima* challenge on juvenile oysters was studied by evaluating the gene-expression patterns of *Cg-p21*, *Cg-CAFp55*, *Cg-EF2*, and *Cg-LGBP*. The transcript levels of these genes were analyzed, firstly, through semi-quantitative RT-PCR to examine the expression patterns of each selected gene throughout the bioassay.

**Figure 2:**
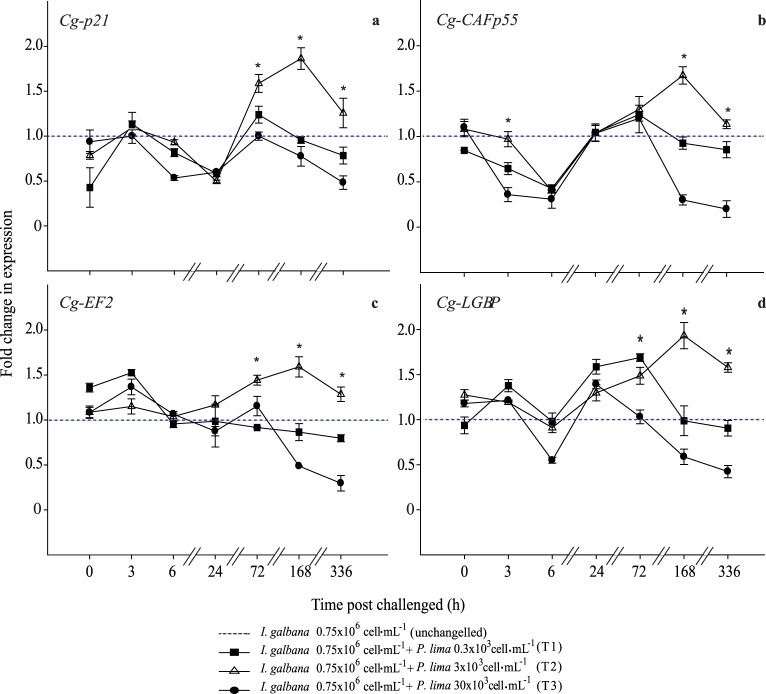
Analysis of differentially expressed genes RT-PCR. *Crassostrea gigas* genes related to cell cycle regulation (a, b, and c) and immune system (d) after challenge with *Prorocentrum lima*. Asterisk indicates significant differences between treatments (*p*<0.05 in Fisheŕs HSD).

The results in our work showed that the analyzed genes exhibited a constitutive expression under normal conditions (the information encoded in each of them was continuously transcribed) but inducible under sub-chronic exposure. The expression levels of the *Cg-p21*, *Cg-CAFp55*, and *Cg-EF2* genes increased in the mixed diet with 3×10^3^ cells mL^−1^; after 72 h, as a compensatory response for homeostasis maintenance, they were modified by exposure time effect to toxic dinoflagellates (Figure 2). The peak of the greatest expression of these genes was observed at 168 h in the same treatment (3×10^3^ cells mL^−1^). At this density the oyster still compensates the damage caused by the dinoflagellate chemical compounds. Sub-chronic exposure to toxic dinoflagellate in the mixed diet with 30×10^3^ cell mL^−1^ generated an adverse metabolic condition in *C. gigas*, which was confirmed by the detection of low expression levels (Figure 2) at 336 h of exposure. Once the experiment ended, all the organisms were fed only with *I. galbana* for 3 days to observe if the damaged oysters were able to recover on their own. However, during this observation period >40% of the organisms died. The hyperphosphorylation process generated by the presence of OA through *P. lima* cells, probably resulted in eliminating some cell cycle control points, which compromised the integrity of the genome and other critical cellular processes on oysters [41].

On the other hand, the *Cg-LGBP* gene expression level showed a significant variation among all treatments starting from 72 h, which indicated *C. gigas* probably recognized the dinoflagellate cells as a pathogen by signaling and activating its immune system. The peak expression was determined at 168 h in the diet of 3×10^3^ cell mL^−1^. The lowest *Cg-LGBP* detection level supposed immune suppression of *C. gigas* fed mixed diet with 30×10^3^ cell mL^−1^ of dinoflagellate on sub-chronic exposure (Figure 2d).

On *C. gigas*, *p21*, *CAFp55*, *EF2*, and *LGBP* gene expressions have been reported as regulated in response to chemical as well as environmental stressors [42], [43], [44], [45], [46], [47]. Coupled reactions of phosphorylation-dephosphorylation have been suggested as one of the central points of the cell cycle control mechanism where key regulators of cell cycle transitions are cyclin-dependent kinases (CDK) [48], [49]. In most organisms, the *p21*, *CAFp55*, and *EF2* gene expressions are classified into dependent pathways for the cellular cycle [50].

*Cg-p21* is a tumor-suppressor gene that controls G1-S phase expression on cell cycle [51]. Under acute exposure (<24 h), the expression fluctuations monitored on oysters were more evident at 3 h, where significant differences (lower than control) among treatments were observed. Expression variation was minimal in the diet of 3×10^3^ cell mL^−1^ while in the diet of 30×10^3^ cell mL^−1^ a significant sub-expression was observed. A first peak of a time-dose-dependent over-expression was observed at 72 h (Figure 2a) later, relative expression levels diminished in all treatments in regard to control. A second expression peak was observed at 168 h, which was greater in 3×10^3^ cell mL^−1^ than in 0.3×10^3^ cell mL^−1^ and 30×10^3^ cell mL^−1^; afterwards, the expression level decreased significantly (p<0.05) but not uniformly among control and treatments (Figure 2a); our results showed that the over-expression of *Cg-p21* is time-dose-dependent.

The *p21* levels in cells are controlled by the tumor-suppressor protein *p53*. Activation of the *p53* signaling pathway is due to DNA-damaging agents, which results as either a cell cycle checkpoint activation to promote cell-survival or an apoptotic cell death [6], [52], [53]. These observations agree with metabolism regulation and cell cycle and gene expression coordination observed at 168 h and 336 h in our work.

The p55 subunit chromatin-assembly factor (*Cg-CAFp55*) is an important part of the special function of histones, involved in genome integrity maintenance and transcriptional regulation [20, 41.]. In our work *Cg-CAFp55* had an early response (3 h exposure) in the mixed diet 3×10^3^ cell mL^−1^ of dinoflagellate (Figure 2b); afterwards, no significant differences (*p*<0.05) were observed between treatments, except when compared with control. From 6 h to 168 h the expression level increased progressively and significantly (*p*<0.05) for 3×10^3^ cell mL^−1^. In treatment of 30×10^3^ cell mL^−1^ of dinoflagellate, the expression drastically decreased from 168 h to the end of the experiment where non-expression was detected. Considering that OA is a potent tumor promoter with aneugenic and clastogenic effects on hereditary material, most notably by DNA breaks and alterations in DNA repair mechanisms [6], [54], recently, the presence of histone variants involved in DNA chromatin repair and specialization has been revealed in mussels and clams based on genotoxicity tests of OA in the marine environment [41].

The *Cg-EF2* involved in the translation process of eukaryotic cells and subjected to phosphorylation is directly associated with peptide-chain elongation stimulation rate [55]. The translation rate is controlled by phosphorylation on several serine and threonine residues [55], [56]; thus *Cg-EF2* phosphorylation state could interfere with the ability of the factor to interact constructively with the ribosome. In our scenario, protein phosphatase inhibitors in the dinoflagellate lead to increased *Cg-EF2* phosphorylation and protein synthesis inhibition. This gene was over-expressed immediately after each *P. lima* dose addition (3 h exposure) on the three treatments. In general terms its expression decreased significantly (*p*<0.05) from 6 h (acute exposure) to 336 h in 0.3×10^3^ cell mL^−1^ (T1) and 30×10^3^ cell mL^−1^ (T3) treatments (sub-chronic exposure). In contrast, in treatment of 3×10^3^ cell mL^−1^ (T2) a gradual expression increase was observed from 6 h to 168 h of exposure; after 168 h a significant (*p*<0.05) decrease on the expression level was observed until the end of the experiment (Figure 2c). Thus, in our study we may presume that the primary effect of OA in inhibiting elongation apparently results from the much greater sensitivity of *Cg-EF2* dephosphorylation.

Nevertheless, our work represents only a small contribution to understanding the mechanisms of the effect of *C. gigas* exposure to *P. lima* as in a natural red tide event. Then, the significant time-dose-dependent expression decrease observed after 168 h suggests that an obvious obstruction in the oyster’s protein synthesis is taking place. In addition the quantified responses of *Cg-p21* and *Cg-CAFp55* also showed low expression levels after 168 h exposure, making a down-regulation in cell cycle progression evident. Thus checkpoints of cell-cycle dysfunction could result in cell death or at least in an increased susceptibility to environmental perturbations such as DNA damaging agents (genotoxics).

On shellfish and other invertebrates, host defense mechanisms and homeostasis are key components modulated by the innate immune system [57]. Organisms distinguish between self and other receptors through recognition, among which lipopolysaccharide-β-1, 3-glucan binding proteins (*LGBP*) stand out [58]. *Cg-LGBP* expression levels are shown graphically in Figure 2d, where significant differences are evident between treatments and control, which indicates that *P. lima* stimulated the *Cg-LGBP* transcription gene on *C. gigas* independently of dinoflagellate cell number.

Under-expression was observed until 336 h in 0.3×10^3^ cell mL^−1^ (T1) and 30×10^3^ cell mL^−1^ (T3); as on all previously presented genes a significant over-expression was observed in 3×10^3^ cell mL^−1^ (T2) from 6 h to 168 h of exposure with a final decrease at 336 h (sub-chronic) of exposure, particularly notable in 3×10^3^ cell mL^−1^ (Figure 2d). Figure 2 shows a consistent drop in the expression level of the genes analyzed in all treatments, whose particular behavior may be associated with two phenomena: temporal shell closure documented in mollusks to evade unfavorable environmental conditions [25] and metabolism reduction to decrease the impact of toxic cells or compounds [32]. For this reason, under our experimental conditions until 3 h exposure, the effect of dinoflagellate presence is barely noticeable that *C. gigas* recognizes *P. lima* as inappropriate food; in fact during this period it is regulated by pseudofeces formation. Only from 12 h exposure was a clear effect of *P. Lima* observed in the expression of the genes analyzed. Additionally, in laboratory bioassays *C. gigas* was observed to select its food from a mixture of microalgae (*I. galbana*, *G. catenatum*, and *P. lima;* Lopez-Cuevas, pers. com.), and *P. lima* began to be effectively filtered from 3 to 6 h exposure; both observations largely explain the behavior observed in the expression of the genes studied in relation to exposure time.

Moreover, contrary to what we expected, a proportional behavior was not observed in *C. gigas* by the effect of *P. Lim*a cell number density. The results obtained can be explained as follows: *P. lima* at a density of 0.3×10^3^ cells mL^−1^ is an imperceptible cell concentration in plain sight in a water body; it has a moderate effect in oysters that does not compromise their survival since only a transient expression increase is observed (24 to 72 h), and then it behaves similarly to the control. In organisms exposed to 30×10^3^ cells mL^−1^ the damage caused by *P. lima* was irreversible; the fall in expression of the genes analyzed in oysters could be interpreted as the beginning of the organisms’ death process [39]. Sar, Ferrairo, and Reguera [59] reported that in the range 0.1×10^3^ to 1×10^3^ cells mL^−1^ of *Dinophysis* and/or *Prorocentrum* (DSP producers) the presence of toxins in bivalve mollusks is detected; then 30×10^3^ cell mL^−1^ *P. lima* is well above this range of toxicity.

### Gene Expression by Real-time Quantitative (RT-qPCR)

**Figure 3:**
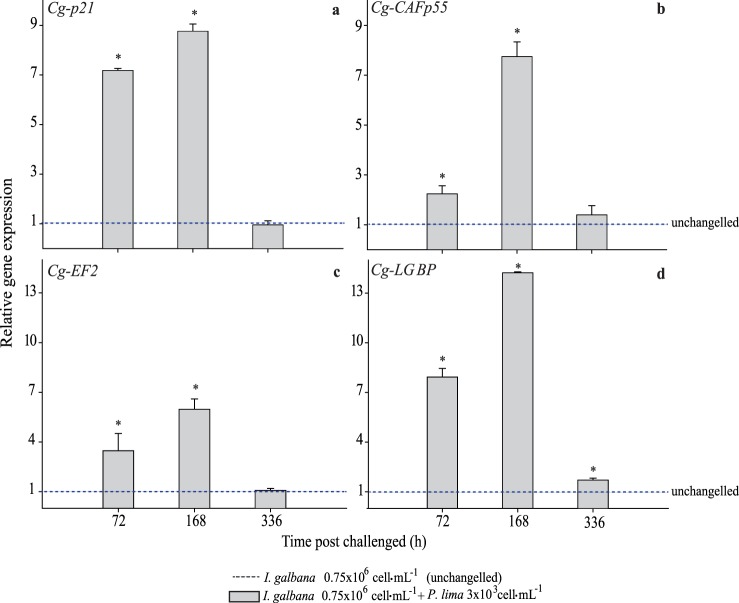
Analysis of relative expression (qPCR). *Crassostrea gigas* genes related to regulation cell cycle (a, b, c) and immune system (d) after challenge to T2 (3×10^3^ cells mL^−1^). Bars represent standard deviation (SD) from the mean value, normalized with *actin*, *tubulin* and *28S ribosomal RNA* and relative to calibrator (dotted line). Asterisk indicates significant differences between treatment and control (*p*<0.05 in Fisheŕs HSD).

Quantitative screening was performed by qPCR for the four genes (*Cg-p21*, *Cg-CAFp55*, and *Cg-EF2*) in sub-chronic-phase (exposure times of 72, 168, and 336 h); we made expression measurements on oysters fed with mixed diet 3×10^3^ cells mL^−1^ of dinoflagellates and those fed nontoxic control (Figure 3). Their expression pattern was similar with those obtained from the semi-quantitative technique. The highest point in transcript level was at 168 h for all genes. Genes related to cell cycle regulation and translation had a high expression principally at 72 and 168 h and dropped at 336 h (Figure 3) showing that oyster cells were affected in their checkpoint cell cycle or apoptotic function by the effect of toxic cells. The consequences could be an abnormal replication control or coordination loss between DNA-replication and cell-cycle progression; in any case, genome instability may result, which is a characteristic of tumor cells [19], [20], [41].

The gene expression level of *Cg-p21*, *Cg-CAFp55*, and *Cg-EF2* increased significantly (*p*<0.05) at 168 h; then the high transcript level found in oysters fed with mixed diet 3×10^3^ cells mL^−1^ of *P lima*, suggest induction at checkpoints making it possible to reach equilibrium.

The *Cg-LGBP* gene was up-regulated from early exposure indicating that it behaves as a constitutive gene, but it is also inducible when exposure conditions (acute and sub-chronic) play a critical role on *C. gigas*-*P. lima* interaction, playing a major function as a pattern recognition receptor.

## Conclusions

On *C. gigas* toxic dinoflagellate ingestion caused a clear modulation of mRNA expression of the genes involved in cell cycle regulation and immune system. An acute exposure caused an alteration of the transcript levels of all the studied genes, which indicates an immediate or early stress response. On the other hand, a sub-chronic exposure generated a higher expression level in all the genes causing a major impact, which could be related to DNA damage and control loss of the cell cycle. This genomic instability might lead to diseases or severe pathologies including death in oysters. The *Cg-LGBP* expression level that increased significantly shows an activation of the innate immune system as first line of defense on *C gigas* against *P. lima* cells or toxins, suggesting it was recognized as a pathogen agent.

While HAB events are much more complex than the bioassays shown here, our study is the first one dealing with understanding and characterizing oysters’ molecular-level responses through the simplification of a red tide phenomenon caused by a DSP-producing organism. More genomic and transcriptome studies should be performed to go more in depth in bivalve mollusks’ molecular responses.

## Methods

### Animals

*C. gigas* (Thunberg 1793) juvenile oysters (3–5 mm) species were obtained from the production laboratory “Acuacultura Robles SPR de RI”, located in Magdalena Bay (Las Botellas), BCS, México. Oysters were acclimated for a minimum period of 7 days in aerated filtered seawater (0.22 µm) at 20°C±1°C and 34 g L^−1^ before their use in experiments. Maintenance diet consisted of a monoalgal diet of *Isochrysis galbana* strain ISG-1 (0.75×10^6^ cells mL^−1^) commonly used as food in laboratory bivalve culture, obtained from the Live Food Laboratory (CIBNOR).

### Microalgae

In this study we used the epibenthonic dinoflagellate *P. lima* (strain PRL-1) isolated from Isla El Pardito (24.5° N, 110.4° W) located in Bahía La Paz on the Gulf of California, BCS, México [36]. The toxin content (under standard culture conditions) was characterized by LC/MS: OA = 2.041 pg cell^−1^, DTX1 = 1.33 pg cell^−1^ and DTX2 = 0.09 pg cell^−1^ [36], [60]. *P. lima* was grown in F/2+Se [61] and prepared with filtered seawater (0.45 µm), UV sterilized in volumes of 1000 mL in 2.8 L polycarbonate Fernbach flasks with 12∶12 h light: dark cycles (irradiance of 150 µM m^2^/s), salinity 35 g L^−1^, at 23°C±1°C until a population density of 30×10^3^ cells mL^−1^ was reached (late exponential phase, day 18). The microalgae *I. galbana* was used as non-toxic diet in the control [62], [63]. Cell concentration in both cultures and feeding experiments were determined by cell counts in Sedgwick–Rafter counting slides and Neubauer chamber (0.1 mm in depth) after fixation with Lugol’s solution [64] (with an optical microscope. For bioassays, cells were harvested by centrifuge (800×*g* for 10 min) on the late exponential growth phase [64] and adjusted to the required concentrations for each case with sterile seawater.

### Experimental Design and Sample Collection

Bioassays were conducted in a temperature-controlled room (22°C±1°C) at a salinity of 35 g L^−1^. Oysters were exposed to three cell densities of toxic dinoflagellate (*P. lima*) for 336 h. Dinoflagellate doses were chosen according to densities observed during HABs on field [36], [60]. For the bioassays, groups of 20 oysters (by triplicate) were exposed to three cell suspensions of *P. lima* (0.3×10^3^, 3×10^3^ and 30×10^3^ cells mL^−1^) combined with a fixed amount of *I. galbana* (0.75×10^6^ cells mL^−1^) as a control, using 100 mL transparent polypropylene containers with a mixture of *P. lima* and *I. galbana* in proportion 1∶1 (50 mL, final volume). Microalgae were provided a single dose each 24 h; aeration was used during feeding experiments to avoid cell sedimentation.

Samples (juvenile oysters) of five organisms each were randomly taken at 0, 3, 6, and 24 h (acute exposure <24 h) and 72, 168, and 336 h (sub-chronic exposure >24 h). Five organisms were extracted from each treatment and transferred to Eppendorf tubes; oysters were washed with sterile seawater, removing excess liquid with adsorbent towels, and frozen immediately in 750 µL of TRIzol (TRIzol Plus RNA Purification Kit, Invitrogen) at −80°C until further processing and analysis.

### Extraction of RNA and First Strand cDNA Synthesis

Samples were thawed on ice and total RNA was extracted with TRIzol® following manufacturer’s instructions. Samples (n = 5) were homogenized using a glass pestle; later, two consecutive TRIzol® extractions were done at each sample. RNA quality was verified by visual inspection of *18S* and *28S ribosomal RNA* bands on agarose-TBE gels. Purity and concentration of nucleic acids were determined by spectrophotometry (Nanodrop 2000, Thermo Scientific) by OD 260/280 and OD260/230 absorbance ratios (range, 1.90–2.08). To ensure complete DNA absence, a direct PCR was done with 1 µL of each RNA preparation using 28S ribosomal specific primers as a no-amplification control. Afterwards, 0.5 µg were used from each verified RNA sample for cDNA synthesis using First-Strand cDNA Synthesis Reaction (Invitrogen®). Total RNA was reverse-transcribed using oligo-dT, and resulting cDNA was stored at −80°C until use.

### Gene Expression Study by RT-PCR

Changes on gene expression were determined by semi-quantitative PCR. Previously, amplicons for each primer set (gene, Table 1) were evaluated by electrophoresis gel (pattern and size) as well as by direct sequencing (Macrogen) to ensure target identity (data not shown). Amplification reactions were performed in triplicate and a negative control (without template) was included in all runs. PCR reactions of genes encoding the cycle regulator p21 protein (*Cg-p21*), chromatin assembly factor 1 P55 subunit (*Cg-CAFp55*), elongation factor 2 (*Cg-EF2*), and lipopolysaccharide/β-1, 3 glucan binding protein (*Cg-LGBP*) were performed in a Corvette Palm thermal cycler in a final volume of 50 µL, containing: 1 µL of cDNA (160 ng), 5 µL of 10×PCR-buffer (Qiagen), 1.5 µL of MgSO_4_ (2.5 mM), 1 µL of each primer (10 pmol), 1 µL of each dNTP (200 mM), and 1.0 U DNA Polymerase (Invitrogen). The amplification program consisted of an initial heat activation step of 95°C/60 s, 35 cycles of 94°C/60 s, 45°C/60 s, and 72°C/1 min, with a final extension step of 72°C/10 min. PCR products were resolved at 80 V in agarose/Synergel®-TBE 1% gels for 1 h; electrophoresis was developed in a submarine system (BioRad). Gels were observed under UV light and digitally documented in a UVITEC system (UVP Inc) under the following standardized conditions: focus 4, magnification 25X, and brightness 0.400. The fluorescence signal quantification (of each amplicon) was done by UVIDOC V. 97 software.

**Table 1:** Combinations of primers used in the expression analysis by RT-PCR.

Gen	Primer
p21 protein (*Cg-p21*)	F5′-CATGTCCTGTATATACATG-3′
	R5′-GATGAACCTCAAATAGAG-3′
Chromatin assembly factor 1 P55 subunit(*Cg-CAFp55*)	F5′-TTCAGACACAGAGCAGA-3′
	R5′-TCGTGGAAGATGTGGC-3′
Elongation factor 2 (*Cg-EF2*)	F5′-GTCAACGAAGGAGGAC-3′
	R5′-ACGACCAGAGCTCCAT-3′
Lipopolysaccharide/β-1, 3glucan binding protein(*Cg-LGBP*)	F5′-CTTGTCATTCCAGGGTT-3′
	R5′-TCTGGCGAAATTGACGT-3′

Forward primer (F), Reverse primer (R).

### Gene Expression by Real-time Quantitative (RT-qPCR)

The quantitative screening was performed by RT-qPCR for the four genes in sub-chronic-phase: *Cg-p21*, *Cg- CAFp55*, *Cg-EF2*, and *Cg-LGBP*. Primers that were not used in previous works were designed from *C. gigas* specific sequences deposited in GenBank with the following characteristics: size from 22–24 bp, Tm 66–69°C, GC content between 48–55%, and amplicon size between 75–150 bp. Sequences and GenBank accession numbers are listed in Table 2. Primers efficiency was tested using the standard curve method. For this purpose, a serial dilution (1∶5) was made from a single cDNA sample consisting of a pool of all cDNA from the different treatments. All primers used showed an efficiency between 1.95 and 2.0 (Table 2). The RT-qPCR analysis was performed in holding Strip Tubes (0.1 mL) (Qiagen) in triplicate, using Rotor gene 6000 Real-Time PCR detection system (Corbette), with a total reaction volume of 15 µL. A qPCR cocktail-mix was carefully prepared in our laboratory. Each reaction mixture had 0.75 µL of 20x EvaGreen fluorescent dye (Biotium), 50 mM MgCl_2_, 2 mM dNTP (each), 0.3 U of platinum Taq DNA polymerase (Invitrogen), 0.05 µM of each primer and 3.2 ng 5 µL^−1^ of cDNA. Amplification conditions were: 95°C 5 min followed by 40 cycles of 95°C for 10 s and 61°C for 60 s and 74°C (10 s) acquiring fluorescence at 79°C (1 s); finally, a dissociation step from 65°C to 95°C (1°C/s) was done. Specificity of the RT-qPCR product was analyzed by a dissociation curve performed after amplification, observing a single peak at the expected Tm. To maintain consistency, the baseline was set automatically by the software. The results were expressed as relative gene expression of transcripts normalized by the set reference genes *actin, β-tubulin* and *28S ribosomal RNA,* using the 2^−(ΔΔ**ct**)^ method [65].

**Table 2:** Genes and primer sequences used for Real-Time Quantitative PCR (qPCR) and GenBank accession numbers.

Primer	Primer sequence (5′-3′)	Gene name	Amplicon	PCR	GenBank
			size (pb)	Efficiency	ref.
Cg- *28S-*F	GGAGTCGGGTTGTTTGAGAATGC	*Ribosomal* *subunit 28S*	114	1.97	AY632555
Cg- *28S-*R	GTTCTTTTCAACTTTCCCTCACGG				
Cg-*tub*-F	AGCAGATGTCGTAGAGAGCTTC	*Tubulin β5*	144	1.96	CB617442
Cg-*tub*-R	TGAACACATTCTCCGTTGTCCC				
Cg-*act*-F	TACTCTTTCACCACCACAGCCG	*Actin (GIA)*	117	1.95	AF026063
Cg-*act*-R	TAGAGATGAGGATGAAGCAGCAG				
Cg-*p21*-F	TTCCCATTCCTCCCATGTTGTTC	*Cell cycle* *regulator p21* *protein*	100	1.98	CB617437
Cg-*p21*-R	ACAGGCGACATGGATTTAGAAGC				
Cg-*CAFP55*-F	TCGAAGATCCCACAAAGCAACAG	*Chromatin* *assembly* *factor 1 p55* *subunit*	77	1.99	CB617555
Cg-*CAFP55*-R	TGTCCTTCAACCCCTACAGCGA				
Cg-*EF2*-F	TTGATCACGGCAAGTCTACTCTG	*Elongation* *factor 2*	109	2.0	CB617558
Cg-*EF2*-R	GAGATGGCAGTGGACTTGATGG				
Cg-*LGBP*-F	TTGTCCAGTTCTCCCAGCTTCC	*Binding* *protein to* *lipopolysaccha* *ride and β 1, 3-* *glucan*	108	1.95	CB617438
Cg-*LGBP*-R	GACACTGGAATGGGATGAAGAAC				

Forward primer (F), Reverse primer (R).

### Statistical Analysis

Following a preliminary data analysis and considering that the 28S gene expression showed the lowest variation with respect to exposure time and dinoflagellate dose, then this gene was used as gene reference for semi-quantitative PCR. Thus, expression data are reported as relative expression values based on densitometry where a value of 1 is equivalent to the gene expression in the basal state (unchallenged oysters fed with *I. galbana*) at different exposure times. A two-way variance analysis (ANOVA) was applied while the RT-qPCR and quantification results were compared by one-way ANOVA, both followed testing normality assumptions of data distribution and variance homogeneity, as well as a Fisheŕs multiple comparison test (α = 0.05); Statistica software ver. 7 was used. The statistical significant difference for the analysis was *p*<0.05.

The analyses were based on the CT values of the quantitative PCR products. The CT was defined as the PCR cycle at which the fluorescence signal crossed a threshold line that was placed in the exponential phase of the amplification curve [65]. The comparative CT method was used to analyze gene expression levels. The CT for the target gene amplification and the CT for the reference genes (*actin, β-tubulin and 28S ribosomal RNA*) were determined for each sample. Differences in CT geometric mean for the target and internal controls called ΔCT were calculated to normalize the data. The control group, called the calibrator, was used as the reference sample. The ΔCT for each sample was subtracted from the ΔCT of the calibrator; the difference was called ΔΔCT value. Gene expression levels could be calculated by the 2^−(ΔΔ**ct**)^ method, and the value stood for an n-fold difference relative to the calibrator [65].
